# Literature Survey and Further Studies on the 3-Alkylation of N-Unprotected 3-Monosubstituted Oxindoles. Practical Synthesis of N-Unprotected 3,3-Disubstituted Oxindoles and Subsequent Transformations on the Aromatic Ring

**DOI:** 10.3390/molecules22010024

**Published:** 2016-12-26

**Authors:** Eszter Kókai, Gyula Simig, Balázs Volk

**Affiliations:** 1 Department of Organic Chemistry and Technology, Budapest University of Technology and Economics, P.O.B. 91, 1521 Budapest, Hungary; ekokai@mail.bme.hu; 2 Directorate of Drug Substance Development, Egis Pharmaceuticals PLC., P.O.B. 100, 1475 Budapest, Hungary; simig.gyula@egis.hu; 3 Department of Materials Technology, GAMF Faculty of Engineering and Computer Science, Pallasz Athéné University, P.O.B. 700, 6001 Kecskemét, Hungary

**Keywords:** oxindole, alkylation, lithiation, regioselectivity, oxidation

## Abstract

The paper provides a comprehensive review of the base-catalysed C3-alkylation of N-unprotected-3-monosubstituted oxindoles. Based on a few, non-systematic studies described in the literature using butyllithium as the deprotonating agent, an optimized method has now been elaborated, via the corresponding lithium salt, for the selective C3-alkylation of this family of compounds. The optimal excess of butyllithium and alkylating agent, and the role of the halogen atom in the latter (alkyl bromides vs. iodides) were also studied. The alkylation protocol has also been extended to some derivatives substituted at the aromatic ring. Finally, various substituents were introduced into the aromatic ring of the N-unprotected 3,3-dialkyloxindoles obtained by this optimized method.

## 1. Introduction

**Figure 1:**
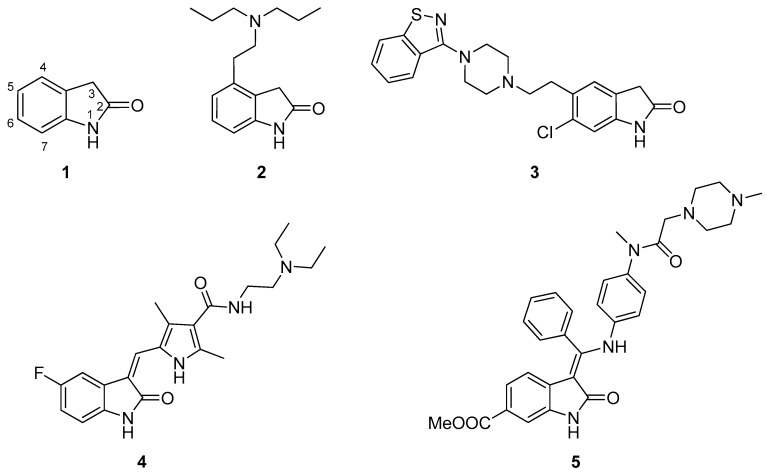
Oxindole (**1**) and marketed drugs with oxindole skeleton (**2**–**5**).

The biological activity of 1,3-dihydro-2*H*-indol-2-one (oxindole (**1**), Figure 1) derivatives and their structural relationship to indoles render these compounds important targets in medicinal and synthetic organic chemistry. Launched drugs possessing an oxindole skeleton are summarized in Figure 1: the dopamine agonist ropinirole (**2**) for the treatment of Parkinson’s disease and restless legs syndrome; the atypical antipsychotic ziprasidone (**3**) and two oncology drugs from the tyrosine kinase inhibitor family, sunitinib (**4**) and the recently launched nintedanib (**5**). Several other compounds have reached human Phase III [1], Phase II [2] or Phase I [3] clinical trials, and thousands of further oxindole derivatives are or were studied in preclinical testing in various therapeutic fields.

**Scheme 1:**
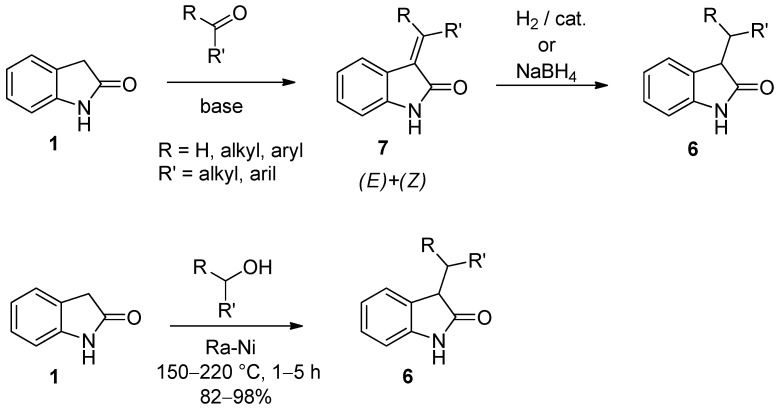
Reductive alkylation reactions of oxindole (**1**) to give 3-alkyloxindoles **6**.

According to the literature, N-unprotected 3-alkyloxindoles **6** can be prepared by condensation of oxindole (**1**) with ketones or aromatic aldehydes and subsequent reduction of the primarily formed 3-alkylideneoxindoles, **7**. However, in the case of aliphatic aldehydes the yields are low because of aldol-type side reactions [4,5,6]. In order to avoid these difficulties, we disclosed an efficient method for the regioselective synthesis of N-unprotected 3-alkyloxindoles **6**, based on the Raney nickel (Ra-Ni) induced 3-alkylation of oxindole (**1**) with primary and secondary alcohols (Scheme 1) [7,8,9].

This reaction involves a reductive alkylation as the key step: Raney nickel acts as the oxidizing agent in the transformation of the alcohol to the corresponding carbonyl compound, then as the catalyst during the reduction of the in situ-formed 3-alkylideneoxindole.

**Scheme 2:**
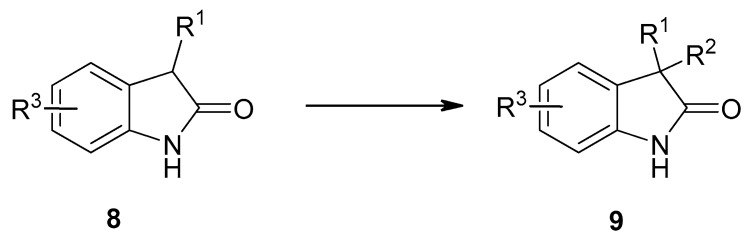
C3-Alkylation of 3-alkyloxindoles **8**.

Next we set ourselves the task to develop an efficient method for the 3-alkylation of N-unprotected 3-alkyloxindoles (**8**, Scheme 2) to give N-unprotected 3,3-dialkyloxindoles **9**.

3-Alkyloxindoles **8** have two regiochemically distinct and easily removable protons (N–H, C3–H), thus rendering possible the formation of N- and C3-alkylated products in a deprotonation-alkylation sequence (i.e. alkylation by nucleophilic substitution). In order to find optimal reaction conditions for the regioselective alkylation of 3-alkyloxindoles **8** we first analyzed the results of systematic studies on the alkylation of oxindole (**1**) described in the literature.

**Scheme 3:**
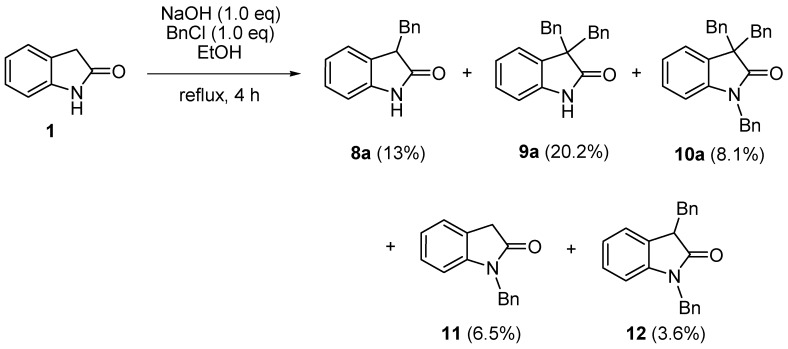
Direct benzylation of oxindole (**1**) in the presence of sodium hydroxide.

In a systematic study described by Gruda in 1972 [10], alkylation of oxindole (**1**) with benzyl chloride was carried out in the presence of sodium bases (NaOEt, NaOH). Besides the 3,3-dibenzyl derivative (**9a**) as the main product (20.2%), 3-benzyl- (**8a**), 1,3,3-tribenzyl (**10a**), 1-benzyl- (**11**) and 1,3-dibenzyloxindole (**12**) products (13%, 8.1%, 6.5% and 3.6%, respectively) were isolated by chromatographic separation (Scheme 3). It was demonstrated that, in addition to C-alkylation, N-alkylation also occurred under these conditions.

**Scheme 4:**
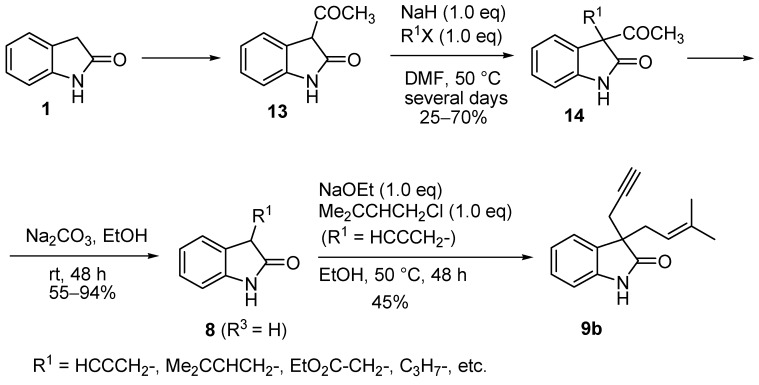
Alkylation of oxindole (**1**) via its sodium salt using a protection-deprotection approach.

A protection-deprotection approach was described by Reisch et al. for the synthesis of 3-monosubstituted oxindoles [11]. 3-Acetyloxindole (**13**), easily available from oxindole (**1**) [12,13], was alkylated in position 3 in low to moderate yield with various alkyl, alkenyl and alkynyl halides(1.0 eq) in the presence of NaH (1.0 eq) (Scheme 4).

The 3-acetyl protecting group increases the acidity of the C3 position, thereby improving selectivity vs. the N1 atom. 3-Alkyl(alkenyl/ alkynyl)-3-acetyloxindoles **14** thus obtained were then hydrolysed with Na_2_CO_3_ in EtOH to give 3-monosubstituted derivatives **8**, thus rendering the introduction of a second alkyl group into position 3 possible. However, only one representative of this family was described: 3-propargyloxindole was reacted with 3,3-dimethylallylchloride in the presence of NaOEt to give the disubstituted congener **9b** in 45% yield. The low yield of **9b** may be due to an incomplete selectivity in this step. Nevertheless, this method has several drawbacks: numerous reaction steps, very long reaction times, chromatographic purifications and low overall yield.

In later studies on the 3-alkylation of 3-monosubstituted oxindoles, sodium hydride (1.0–1.1 eq) was used again as the base in the alkylation reaction of 5-fluoro-3-methyloxindole with 2-bromoethyl methyl ether (1.0 eq, 19% yield after purification by high performance liquid chromatography (HPLC) [14], in the 3-methylation (with 2.9 eq MeI) of 3-arylated 7-fluorooxindole (64% yield, 93% purity) [15], and in the reaction of 3-cyclopropyl- or 3-cyclobutyloxindole with MeI (1.0 eq, chromatographic purification, undisclosed yield) [16]. Thus, sodium bases seem to be unsuitable for the high-yielding selective introduction of a second substituent in position C3.

Potassium bases and cesium carbonate also proved to be disadvantageous in several attempted variants: *t*-BuOK in DMF [17], *t*-BuOK in tetrahydrofuran (THF) [18], potassium bis(trimethylsilyl)amide in THF [19], KI-K_2_CO_3_ in acetone [20,21], KI-K_2_CO_3_ in THF [21], K_2_CO_3_ in dimethyl acetamide [22,23], and Cs_2_CO_3_ in DMF [24].

**Scheme 5:**
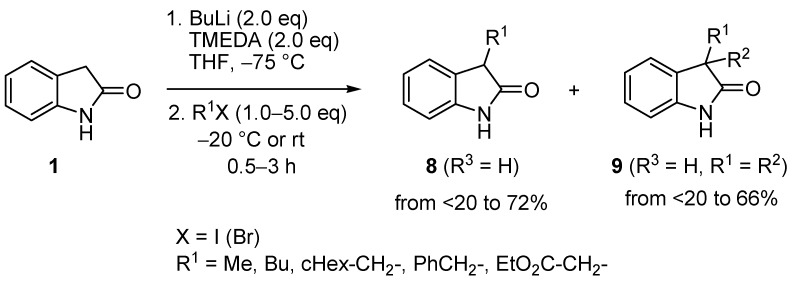
Direct alkylation of oxindole (**1**) via its lithium salt.

Use of a lithium base, first described by Kende et al., proved to be a better approach towards selective C3-alkylation [25], Treatment of oxindole (**1**) with butyllithium (BuLi, 2.0 eq) in the presence of *N*,*N*,*N′*,*N′*-tetramethylethylenediamine (TMEDA, 2.0 eq) in THF at −75 °C followed by reaction with various alkyl halides at a temperature between −20 °C and room temperature gave varying yields of the 3-monosubstituted (**8**, from <20% to 72% yield) and 3,3-disubstituted (**9**, from <20% to 66% yield) products (Scheme 5), the ratio of which depended also on the excess of alkylating agent and on the reaction conditions. Besides the pleasing lack of N-alkylation, the major drawbacks of this BuLi-TMEDA protocol are the limited mono/di selectivity, the need for chromatographic purification, and the fact that bromides (except for benzyl bromide and ethyl bromoacetate) were unreactive, therefore the corresponding iodides, which are less easily available and more expensive, had to be used. Two examples are given also for the second alkylation in position 3 under the same conditions: 3-butyloxindole was alkylated with iodomethylcyclohexane (74% yield) and 3-benzyloxindole with benzyl bromide (87%). It is noteworthy that omission of TMEDA gave poor results in all these reactions.

As demonstrated above, deprotonation with lithium bases offers a more efficient approach towards selective C3-alkylation than alkali metal bases. Since the first step is the formation of a N,C-dianion, attempts for the selective 3-alkylation of 3-monosubstituted oxindoles **8** with less than 2.0 eq BuLi are always low-yielding. Accordingly, 3-ethylation of 3-methyloxindole (1.0 eq BuLi, 3 eq LiCl, 5.0 eq EtI, THF) [26] or 3-propargylation of 3-ethyloxindole (1.2 eq BuLi, 1.0 eq propargyl bromide, THF) [27] gave, after chromatographic purification, 25% and 55% yields, respectively. Use of 2.0 eq BuLi led to better results: 3-methyloxindole was 3-methylated (2.0 eq BuLi, 9.4 eq MeI) in 75% yield after chromatographic purification [28].

The BuLi-TMEDA protocol described by Kende [25] and its slight modifications (2.0–2.5 eq BuLi, 2.0–3.0 eq TMEDA, 1.0–2.2 eq alkyl iodide, THF, −25 °C or −78 °C → r.t.) have been applied by various research groups for the 3-alkylation of 3-monosubstituted oxindoles **8**. Nevertheless, in most cases the yields are not disclosed [16,29,30], or low [31,32,33]. Only a few successful applications are described. 3-Methyl-6-methoxyoxindole was 3-methylated with methyl iodide (MeI, 82% yield after flash chromatography) under similar conditions [34]. Instead of alkyl iodides, Jiang et al. used ethyl 2-bromoacetate as the alkylating agent with 60% yield [35].

Our comprehensive literature search did not reveal further systematic studies (other than those demonstrated in Scheme 3, Scheme 4 and Scheme 5) on the synthesis of N-unprotected 3,3-disubstituted oxindoles **9** starting from 3-monosubstituted oxindoles (**8**). As shown above, due to the biological importance of oxindole derivatives, several research groups applied the above alkylation reactions for the introduction of a second substituent in position 3 of a 3-monosubstituted oxindole **8**, albeit, in quite an erratic way.

**Scheme 6:**
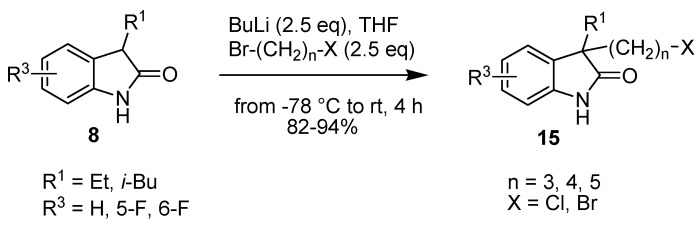
Synthesis of 3-alkyl-3-(ω-haloalkyl)oxindoles.

In the course of our earlier medicinal chemistry studies, selective C3‑alkylation of a small number of 3-alkyloxindole derivatives **8** using α,ω‑dihaloalkanes (2.5 eq) was successfully carried out after deprotonation with BuLi (2.5 eq), resulting in 3-alkyl-3-(ω-haloalkyl)oxindole intermediates (**15**, Scheme 6) [36,37].

For the sake of completeness it is worth mentioning that, due to the ambident nucleophile character of oxindoles, deprotonation and alkylation can take place not only on C3 or N1, but also on the O2 atom. To the best of our knowledge, C,O- or C,N-dialkylations are not described. On the other hand, selective O-alkylation can take place under certain conditions, although the occurrence of this reaction in the scientific literature is very rare. It can only be carried out using special alkylating agents, e.g., trialkyloxonium tetrafluoroborates [38,39,40,41,42,43,44,45,46].

**Scheme 7:**
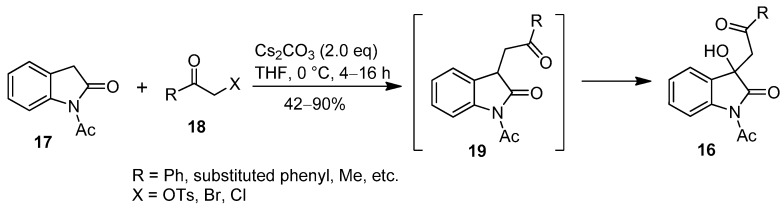
Synthesis of 1-acetyl-3-hydroxy-3-phenacyloxindole derivatives **16**.

Apart from regioselectivity issues, a further difficulty during the alkylation of oxindole derivarives is caused by the observation that position 3 of 3-monoalkyloxindoles **8** is prone to oxidation under basic conditions. Bai et al. described the synthesis of a wide range of 1-acetyl-3-hydroxy-3-phenacyloxindole derivatives **16** starting from 1-acetyloxindole (**17**, Scheme 7) and α-tosyloxyacetophenone (**18**, R=Ph, X=OTs) in an open vial [47]. In a control experiment, the reaction of **17** and α-tosyloxyacetophenone was carried out under nitrogen atmosphere for 8 h, and the 3-monosubstituted oxindole **19** was obtained (yield is not disclosed). Then the reaction was continued (8 h) by opening the flask, leading to the formation of 3-hydroxy derivative **16**, presumably via the corresponding hydroperoxide, the presence of which was proved by electrospray ionization mass spectrometry (ESI-MS). Since the key factor of the suppression of side reactions is the exclusion of atmospheric oxygen, the reductive method elaborated by our research group (Scheme 1) [9] for the synthesis of 3-monoalkyloxindoles **8** is particularly advantageous.

## 2. Results and Discussion

In the present work we set ourselves the task to carry out a deeper study on the scope and limitations of the deprotonation of 3-monosubstituted oxindoles with BuLi and subsequent alkylation. The advantages of using lithium bases (alkyllithiums, lithium dialkylamides) instead of other alkali metal bases in C-alkylation reactions for deprotonation of C-H acids (e.g., ketones, esters, amides) is well documented [48]. Lithium cation, as the smallest alkali metal ion has a stronger tendency to form O–Li and N–Li bonds with increased covalent character [49], thus inhibiting undesirable O- and N-alkylations. 3-Monosubstituted oxindoles **8**, optionally substituted on the aromatic ring, as the starting materials of the present study were synthesized from the corresponding isatins [50].

**Scheme 8:**
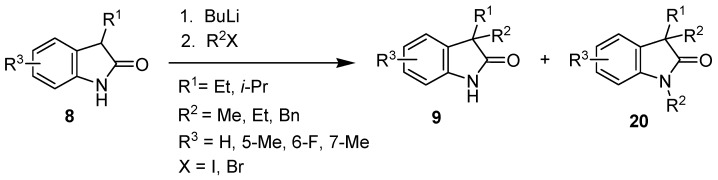
Alkylation of 3-ethyloxindoles **8b**–**e** and 3-isopropyloxindole (**8f**) with various alkyl halides.

Based on the lithiation-alkylation protocol using BuLi (2.5 eq) and α,ω-dihaloalkanes (2.5 eq) described earlier [36,37], we now aimed at the optimization and application of this method to alkylation with alkyl halides. First, 3-ethyloxindole (**8b**, Scheme 8) was used as the model compound for the introduction of a second alkyl group. Despite the complete regioselectivity found in our earlier studies with α,ω-dibromo- and- α-bromo-ω-chloroalkanes [36,37], reaction of **8b** with BuLi (2.5 eq) and MeI (2.5 eq) led to a mixture (Table 1, entry 1) of 3-ethyl-3-methyloxindole (**9c**, 28%) and 3-ethyl-1,3-dimethyloxindole (**20a**, 55%).

This finding is particularly surprising in the light of the paper of Kende et al. that does not describe N-alkylation side-reactions with alkyl iodides [25]. When using decreased excesses of the reagents (2.2 eq BuLi, 1.2 eq MeI), 3,3-dialkyl product **9c** was obtained in 71% yield, while the formation of 1,3,3-trialkyl derivative **20a** could not be detected (entry 2). Unexpectedly, alkylation of **8b** occurred regioselectively even with 2.5 eq of EtI (73%, entry 3). Change of EtI (2.5 eq) to EtBr (1.2 eq) led to even better results (90%, entry 4), and the reaction was also performed with BnBr (80%, entry 5). Extension of the ethylation (with 1.2 eq EtBr) to derivatives **8b**–**e** substituted on the aromatic ring was also successful (entries 6–8).

Reactions of 3-isopropyloxindole (**8f**) gave similar results. Alkylation with 2.5 eq MeI was not regioselective, and led to a mixture of 3-isopropyl-3-methyloxindole (**9i**, 40%, Table 1 entry 9) and 3-isopropyl-1,3-dimethyloxindole (**20b**, 35%), but use of 1.2 eq MeI afforded uniformly **9i** (entry 10). 3-Alkylations could be carried out in a regioselective manner with 1.2 eq EtBr and 1.2 eq BnBr to give the corresponding 3-ethyl (**9j**) and 3-benzyl (**9k**) derivatives in 65% and 63% yields, respectively (entries 11–12).

In order to study the role of TMEDA (applied by Kende et al. during the 3-alkylation reactions of oxindole, **1**) [25], a control experiment (analogous to that shown in entry 4 of Table 1) was performed with BuLi/TMEDA (2.2/2.2 eq) using 3-ethyloxindole (**8b**) as the starting material and ethyl bromide (1.2 eq) as the alkylating agent. The reaction gave practically the same yield (88% vs. 90%) therefore, considering also that the elimination of TMEDA necessitates extra steps during the work-up of the reaction mixture, it was not applied in our standard alkylation protocol.

**Scheme 9:**
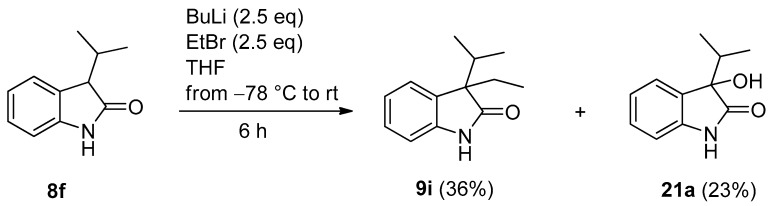
Ethylation of 3-isopropyloxindole (**8f**) under insufficiently inert conditions.

The reactions summarized in Table 1 were strictly performed under inert atmosphere. Prior to the reactions, the flask was made inert by using three consecutive vacuum–argon cycles, and an argon atmosphere was maintained until quenching the reaction. However, during the first series of experiments where a less strict pre-inertization was used, significant amounts of the 3-hydroxy side products were isolated. When starting from 3-isopropyloxindole (**8f**), the alkylation using 2.5 eq BuLi and 2.5 eq EtBr provided, after work-up a significant amount (23%) of 3-hydroxy-3-isopropyloxindole (**21a**, Scheme 9), besides the expected 3-ethyl-3-isopropyloxindole (**9i**, 36%).

**Scheme 10:**
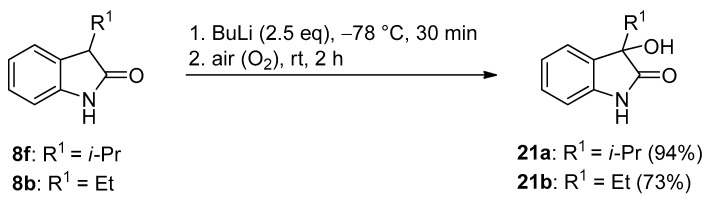
3-Hydroxylation of 3-alkyloxindoles **8b**,**f**.

The targeted synthesis of the 3-hydroxy derivatives **21a**,**b** was carried out with BuLi (2.5 eq) without alkylating agent and under non-inert conditions with good yields (Scheme 10). The presence of the hydroxy moiety in products **21a**,**b** renders further functionalizations possible thereby making these compounds valuable synthetic building blocks.

**Scheme 11:**
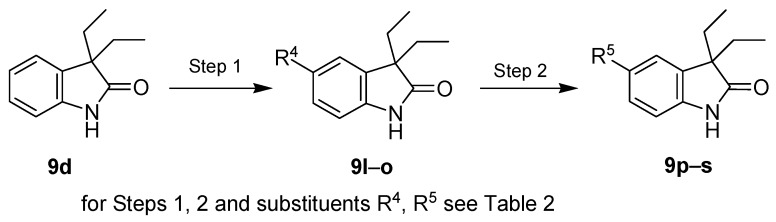
Various functionalizations of 3,3-diethyloxindole (**9d**) on the aromatic ring.

As demonstrated among others by two marketed drugs, ziprasidone (**3**) and sunitinib (**4**), and some further drug candidates [51], substitution at position 5 of the aromatic ring is of importance in the oxindole family. A possible approach for the synthesis of 5-substituted 3,3-dialkyloxindoles is 3-alkylation of a 5-subsituted 3-monoalkyloxindole. Nevertheless, several moieties (e.g., Br, NO_2_, *ortho*-directing groups, etc.) can be incompatible with BuLi-mediated 3-alkylation. Therefore, aromatic substitution reactions were carried out, starting from 3,3-diethyloxindole (**9d**) as the model compound (Scheme 11). Moreover, further modifications of the primarily obtained compounds **9l**–**o** have also been envisaged.

First, reaction of **9d** with sulfuryl chloride in glacial acetic acid at 10–15 °C was carried out to give the 5-chloro congener **9l** in 73% yield (Table 2, entry 1). Bromination of **9d** was performed with bromine and KBr in aqueous dioxane at 90 °C to give 5-bromo derivative **9m** (entry 2) in almost quantitative yield. Nitration of **9d** in a mixture of concentrated sulfuric acid and concentrated nitric acid at 0 °C (entry 3) led to 3,3-diethyl-5-nitrooxindole **9n**, which was reduced by catalytic hydrogenation on palladium on charcoal to 5-amino derivative **9p**. Treatment of starting material **9d** with sulfurochloridic acid afforded 5-chlorosulfonyl derivative **9o** in 98% yield (entries 4–6). This latter was reacted with ammonia (entry 4), *tert*-butylamine (entry 5) or morpholine (entry 6) to give the corresponding aromatic sulfonamides **9q**–**s**.

**Scheme 12:**
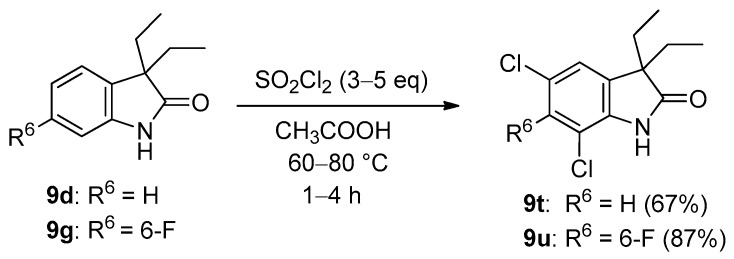
Synthesis of 5,7-dichloro derivatives **9t,u**.

Reaction of 3,3-diethyloxindole (**9d**) with sulfuryl chloride in glacial acetic acid at 10–15 °C led to selective 5-chlorination. However, at elevated temperatures (60–80 °C), in accordance with our earlier observations with 3-ethyl-3-(ω-haloalkyl)oxindoles **15** [36,37], the 5,7-dichloro derivative **9t** was obtained (Scheme 12). Similar chlorination of 3,3-diethyl-6-fluorooxindole **9g** resulted in 5,7-dichloro-3,3-diethyl-6-fluorooxindole (**9u**).

## 3. Experimental Section

### 3.1. General Information

All melting points were determined on a Büchi 535 capillary melting point apparatus (Büchi, Flawil, Switzerland) and on an OptiMelt Automated Melting Point System by Stanford Research Systems (Sunnyvale, CA, USA). IR spectra were obtained on a IFS-113v FT spectrometer (Bruker, Billerica, MA, USA). ^1^H-NMR and ^13^C-NMR spectra were recorded on a Bruker Avance III (400 and 100 MHz for ^1^H- and ^13^C-NMR spectra, respectively) or a Bruker Avance III HD 600 (600 and 150 MHz for ^1^H and ^13^C-NMR spectra, respectively) spectrometer (Bruker, Billerica, MA, USA). CDCl_3_, DMSO-*d*_6_ or CD_3_CN was used as the solvent and tetramethylsilane (TMS) as internal standard. Chemical shifts (δ) and coupling constants (*J*) are given in ppm and in Hz, respectively. The electron ionization (EI) mass spectra were recorded on a Clarus 560 D mass spectrometer coupled with a Clarus 500 gas chromatograph (Perkin-Elmer, Waltham, MA, USA). The ESI+ mass spectra (MS) were recorded on a LTQ XL mass spectrometer (Thermo Fisher, Waltham, MA, USA) coupled with an Acquity^TM^ UPLC (Waters, Milford, MA, USA). Elemental analyses (EA) were performed on a 2400 analyzer (Perkin-Elmer, Waltham, MA, USA) on a VARIO EL III Model CHN elemental analyzer (Elementar, Langenselbold, Germany) or on an Elementar Vario MICRO cube (CHNS) elemental analyzer (Elementar, Langenselbold, Germany). The chloride and bromide contents were determined by titration. The reactions were followed by analytical thin layer chromatography on silica gel 60 F_254_ (Merck, Darmstadt, Germany). All unspecified reagents were purchased from commercial sources. Compounds **9a**–**b**, **9d**, **20a**–**b**, **21a** are known in the literature. Compounds **9c**, **9m**, **9n**, **9p**, **21b** are mentioned but poorly characterized in the literature, therefore their full characterization is given below. Compounds **9e**–**l**, **9o**, **9q**–**u** are new and characterized below.

### 3.2. General Procedure I for the Synthesis of Compounds ***9c**–**h*** (and By-Product ***20a***)

To a mixture of butyllithium in hexane (2.2**–**2.5 eq, 1.6 M) and THF, the solution of the appropriate 3-alkyloxindole **8b**–**e** in THF was added dropwise at −78 °C under argon atmosphere. Then the appropriate alkyl halide (1.2**–**2.5 eq) in THF was added dropwise, the acetone-dry ice bath was removed and the reaction mixture was allowed to warm to room temperature. The stirring was continued for further 4 h, the mixture was quenched with ethanol (EtOH) and the solvents were evaporated. The residue crystallized upon treatment with water. It was triturated in water, filtered, washed with water until the pH was adjusted to 7, then washed twice with diisopropyl ether (DIPE) and dried. Analytical samples were obtained by recrystallization from the indicated solvents.

*3-Ethyl-3-methyl-1,3-dihydro-2H-indol-2-one* (**9c**) [52]. Method A: This compound was prepared according to the general procedure I using BuLi (4.26 mL, 6.82 mmol, 2.2 eq) in THF (3 mL), **8b** (500 mg, 3.10 mmol) dissolved in THF (4 mL), and MeI (232 µL, 528 mg, 3.72 mmol, 2.2 eq) in THF (2 mL). The reaction was quenched with EtOH (1 mL). The product **9c** was obtained as pale yellow crystals (386 mg, 71%), m.p. 143–144 °C (hexane-EtOAc), lit. [52] m.p. 143–145 °C. ^1^H-NMR (600 MHz, CDCl_3_) δ 8.32 (br s, 1H), 7.21 (dt, *J* = 7.6, 1.1 Hz, 1H), 7.16 (d, *J* = 7.3 Hz, 1H), 7.05 (dt, *J* = 7.5, 0.9 Hz, 1H), 6.92 (dt, *J* = 7.5, 0.9 Hz, 1H), 1.96–1.93 (m, 1H), 1.81–1.76 (m, 1H), 1.39 (s, 3H), 0.65 (t, *J* = 7.4 Hz, 3H). ^13^C-NMR (150 MHz, CDCl_3_) δ 183.1, 140.5, 134.3, 127.6, 122.9, 122.4, 109.6, 49.4, 31.4, 23.4, 8.9. IR (KBr) ν 1710, 1618 cm^−1^. MS (ESI): 176.01 [M + H]^+^. EA Calcd. for C_11_H_13_NO (175.23): C, 75.40%; H, 7.48%; N, 7.99%. Found: C, 75.69%; H, 7.36%; N, 7.96%. Method B: This compound was prepared according to the general procedure I using BuLi (4.84 mL, 7.75 mmol, 2.5 eq) in THF (4 mL), **8b** (500 mg, 3.10 mmol) dissolved in THF (4 mL), and MeI (0.48 mL, 1.10 g, 7.75 mmol, 2.5 eq) in THF (2 mL). The reaction was quenched with EtOH (2 mL). The residual oil was purified by gradient elution column chromatography using hexane and ethyl acetate (EtOAc) as the eluents to give **9c** (150 mg, 28%) as colorless crystals.

*3-Ethyl-1,3-dimethyl-1,3-dihydro-2H-indol-2-one* (**20a**) [53]. This compound was prepared according to the general procedure I using BuLi (4.84 mL, 7.75 mmol, 2.5 eq) in THF (4 mL), **8b** (500 mg, 3.10 mmol) dissolved in THF (4 mL), and MeI (0.48 mL, 1.10 g, 7.75 mmol, 2.5 eq) in THF (2 mL). The reaction was quenched with EtOH (2 mL). The residual oil was purified by gradient elution column chromatography using hexane and EtOAc as the eluents to give **20a** (320 mg, 1.69 mmol, 55%) as a yellow oil.

*3,3-Diethyl-1,3-dihydro-2H-indol-2-one* (**9d**) [54]. Method A: This compound was prepared according to the general procedure I using BuLi (9.70 mL, 15.50 mmol, 2.5 eq) in THF (10 mL), **8b** (1.00 g, 6.20 mmol) dissolved in THF (8 mL), and ethyl iodide (1.25 mL, 2.71 g, 15.50 mmol, 2.5 eq) in THF (3 mL). The reaction was quenched with EtOH (2 mL). The product **9d** was obtained as colorless crystals (855 mg, 73%), m.p. 158–159 °C (hexane–EtOAc), lit. [54] m.p. 166–168 °C. Method B: This compound was prepared according to the general procedure I using BuLi (344 mL, 0.55 mol, 2.2 eq) in THF (100 mL), **8b** (40.25 g, 0.25 mol) dissolved in THF (300 mL), and ethyl bromide (22.40 mL, 32.69 g, 0.30 mol, 1.2 eq) in THF (50 mL). The reaction was quenched with EtOH (20 mL). The product **9d** was obtained as colorless crystals (42.60 g, 90%).

*3-Benzyl-3-ethyl-1,3-dihydro-2H-indol-2-one* (**9e**). This compound was prepared according to the general procedure I using BuLi (4.26 mL, 6.82 mmol, 2.2 eq) in THF (3 mL), **8b** (500 mg, 3.10 mmol) dissolved in THF (4 mL), and benzyl bromide (442 µL, 636 mg, 3.72 mmol, 1.2 eq) in THF (2 mL). The reaction was quenched with EtOH (1 mL). The product **9e** was obtained as colorless crystals (626 mg, 80%), m.p. 124–125 °C (hexane–EtOAc). ^1^H-NMR (400 MHz, CDCl_3_) δ 7.66 (br s, 1H), 7.15–7.11 (m, 2H), 7.05–7.01 (m, 4H), 6.90–6.87 (m, 2H), 6.69–6.67 (m, 1H), 3.13 (d, *J* = 13.04 Hz, 1H), 3.04 (d, *J* = 13.05 Hz, 1H), 2.12–2.07 (m, 1H), 1.94–1.89 (m, 1H), 0.66 (t, *J* = 7.4 Hz, 3H). ^13^C-NMR (100 MHz, CDCl_3_) δ 181.1, 141.0, 136.0, 131.4, 130.0, 127.7, 127.6, 126.4, 123.9, 122.0, 109.3, 55.7, 43.8, 30.5, 8.7. IR (KBr) ν 1717, 1474 cm^−1^. MS (ESI): 252.14 [M + H]^+^. EA Calcd. for C_17_H_17_NO (251.33): C, 81.24%; H, 6.82%; N, 5.57%. Found: C, 80.88%; H, 6.78%; N, 5.57%.

*3,3-Diethyl-5-methyl-1,3-dihydro-2H-indol-2-one* (**9f**). This compound was prepared according to the general procedure I using BuLi (8.25 mL, 13.20 mmol, 2.2 eq) in THF (5 mL), **8c** (1.05 mg, 6.00 mmol) dissolved in THF (7 mL), and ethyl bromide (537 µL, 784 mg, 7.20 mmol, 1.2 eq) in THF (4 mL). The reaction was quenched with EtOH (2 mL). The product **9f** was obtained as colorless crystals (930 mg, 76%), m.p. 155–156 °C (hexane–EtOAc). ^1^H-NMR (400 MHz, CDCl_3_) δ 9.53 (br s, 1H), 6.99 (dd, *J* = 7.9, 1.0 Hz, 1H), 6.92 (s, 1H), 6.84 (d, *J* = 7.8 Hz, 1H), 2.33 (s, 3H), 1.96–1.82 (m, 2H), 1.81–1.75 (m, 2H), 0.64 (t, *J* = 7.4 Hz, 6H). ^13^C-NMR (100 MHz, CDCl_3_) δ 183.3, 139.3, 132.4, 131.5, 127.8, 123.6, 109.3, 54.9, 30.6, 21.1, 8.6. IR (KBr) ν 3170, 1695 cm^−1^. MS (EI): 203 [M], 175, 174, 156, 146, 130. EA Calcd. for C_13_H_17_NO (203.29): C, 76.81%; H, 8.43%; N, 6.89%. Found: C, 76.95%; H, 8.24%; N, 7.06%.

*3,3-Diethyl-6-fluoro-1,3-dihydro-2H-indol-2-one* (**9g**). This compound was prepared according to the general procedure I using BuLi (46.06 mL, 73.70 mmol, 2.2 eq) in THF (30 mL), **8d** (6.00 g, 33.50 mmol) dissolved in THF (45 mL), and ethyl bromide (3.00 mL, 4.38 g, 40.20 mmol, 1.2 eq) in THF (10 mL). The reaction was quenched with EtOH (12 mL). The product **9g** was obtained as colorless crystals (5.31 g, 77%), m.p. 149–151 °C (hexane–EtOAc). ^1^H-NMR (400 MHz, CDCl_3_) δ 8.84 (br s, 1H), 7.04 (dd, *J* = 8.2, 5.3 Hz, 1H), 6.74 (ddd, *J* = 9.6, 8.2, 2.4 Hz, 1H), 6.69 (dd, *J* = 8.8, 2.4 Hz, 1H), 1.97–1.88 (m, 2H), 1.83–1.73 (m, 2H), 0.64 (t, *J* = 7.4 Hz, 6H). ^13^C-NMR (100 MHz, CDCl_3_) δ 183.1, 162.4 (d, *J* = 243.8 Hz), 142.6 (d, *J* = 11.8 Hz), 127.7, 123.9 (d, *J* = 9.5 Hz), 108.6 (d, *J* = 22.1 Hz), 98.2 (d, *J* = 27.5 Hz), 54.6, 30.6, 8.6. IR (KBr) ν 3128, 1722, 1140 cm^−1^. MS (EI): 207 [M], 178, 160, 150, 135, 108. EA Calcd. for C_12_H_14_FNO (207.25): C, 69.55%; H, 6.81%; N, 6.76%. Found: C, 69.19%; H, 6.99%; N, 6.92%.

*3,3-Diethyl-7-methyl-1,3-dihydro-2H-indol-2-one* (**9h**). This compound was prepared according to the general procedure I using BuLi (3.92 mL, 6.28 mmol, 2.2 eq) in THF (2 mL), **8e** (500 mg, 2.85 mmol) dissolved in THF (9 mL), and ethyl bromide (255 µL, 373 mg, 3.42 mmol, 1.2 eq) in THF (2 mL). The reaction was quenched with EtOH (1 mL). The product **9h** was obtained as colorless crystals (380 mg, 66%), m.p. 120–121 °C (hexane). ^1^H-NMR (400 MHz, CDCl_3_) δ 8.21 (br s, 1H), 7.03–7.01 (m, 1H), 6.99**–**6.94 (m, 2H), 2.28 (s, 3H), 1.97–1.88 (m, 2H), 1.83–1.74 (m, 2H), 0.63 (t, *J* = 7.4 Hz, 6H). ^13^C-NMR (100 MHz, CDCl_3_) δ 182.4, 140.0, 132.0, 128.9, 122.3, 120.5, 118.4, 55.1, 30.7, 16.5, 8.7. IR (KBr) ν 2966, 1698 cm^−1^. MS (EI): 203 [M], 175, 174, 156, 146, 130, 115. EA Calcd. for C_13_H_17_NO (203.29): C, 76.81; H, 8.43; N, 6.89%. Found: C, 76.87; H, 8.33; N, 7.07%.

### 3.3. General Procedure II for the Synthesis of Compounds ***9i**–**k*** (and By-Product ***20b***)

To a mixture of BuLi in hexane (2.2**–**2.5 eq, 1.6 M) and THF, the solution of 3-isopropyloxindole (**8f**) in THF was added dropwise at −78 °C, under argon atmosphere. Then the appropriate alkyl halide (1.2–2.5 eq) in THF was added dropwise, the acetone-dry ice bath was removed and the reaction mixture was allowed to warm to room temperature. The stirring was continued for further 4–6 h. The mixture was quenched with EtOH (2 mL), saturated ammonium chloride solution (10 mL) was added, then it was stirred for 30 min. The layers were separated and the aqueous layer was extracted with EtOAc (3 × 10 mL). The combined organic layer was washed with brine (10 mL), dried over anhydrous MgSO_4_, filtered and the solvent was removed in vacuo at 40 °C. The residue crystallized upon treatment with hexane (5 mL), then it was filtered. Analytical samples were obtained by recrystallization from the indicated solvents.

*3-Isopropyl-3-methyl-1,3-dihydro-2H-indol-2-one* (**9i**). Method A: This compound was prepared according to the general procedure II using BuLi (4.46 mL, 7.13 mmol, 2.5 eq) in THF (2 mL), **8f** (500 mg, 2.85 mmol) dissolved in THF (6 mL), and MeI (213 µL, 485 mg, 3.42 mmol, 1.2 eq) in THF (2 mL). The product **9i** was obtained as colorless crystals (300 mg, 56%), m.p. 126–127 °C (hexane–EtOAc). ^1^H-NMR (600 MHz, CDCl_3_) δ 8.08 (br s, 1H), 7.26–7.18 (m, 2H), 7.02 (dt, *J* = 7.6, 1.0 Hz, 1H), 6.90 (d, *J* = 7.7 Hz, 1H), 2.13 (sp, *J* = 6.7 Hz, 1H), 1.40 (s, 3H), 1.02 (d, *J* = 7.0 Hz, 3H), 0.79 (d, *J* = 7.7 Hz, 3H). ^13^C-NMR (150 MHz, CDCl_3_) δ 183.0, 140.5, 133.5, 127.5, 123.8, 122.1, 109.4, 52.0, 35.4, 21.5, 17.5, 17.1. IR (KBr) ν 3185, 1717, 1670 cm^−1^. MS (ESI): 190.06 [M + H]^+^. EA Calcd. for C_12_H_15_NO (189.26): C, 76.16; H, 7.99; N, 7.40%. Found: C, 75.89; H, 7.72; N, 7.61%. Method B: This compound was prepared according to the general procedure II using BuLi (4.46 mL, 7.13 mmol, 2.5 eq) in THF (2 mL), **8f** (500 mg, 2.85 mmol) dissolved in THF (6 mL), and MeI (444 µL, 1012 mg, 7.13 mmol, 2.5 eq) in THF (2 mL). The residual oil was purified by gradient elution column chromatography using hexane and EtOAc as the eluents to give **9i** (216 mg, 40%) as colorless crystals.

*3-Isopropyl-1,3-dimethyl-1,3-dihydro-2H-indol-2-one* (**20b**) [55]. This compound was prepared according to the general procedure II using BuLi (4.46 mL, 7.13 mmol, 2.5 eq) in THF (2 mL), **8f** (500 mg, 2.85 mmol) dissolved in THF (6 mL), and MeI (444 µL, 1012 mg, 7.13 mmol, 2.5 eq) in THF (2 mL). The residual oil was purified by gradient elution column chromatography using hexane and EtOAc as the eluents to give **20b** (200 mg, 35%) as yellow oil, lit. [55] m.p. 54 °C. Spectral data are identical with those described in the literature [55].

*3-Ethyl-3-isopropyl-1,3-dihydro-2H-indol-2-one* (**9j**). This compound was prepared according to the general procedure II using BuLi (7.85 mL, 12.55 mmol, 2.2 eq) in THF (8 mL), **8f** (1.00 g, 5.70 mmol) dissolved in THF (12 mL), and ethyl bromide (0.51 mL, 0.75 g, 6.84 mmol, 1.2 eq) in THF (2 mL). The product **9j** was obtained as colorless crystals (0.76 mg, 65%), m.p. 103–104 °C (hexane). ^1^H-NMR (600 MHz, CDCl_3_) δ 8.54 (br s, 1H), 7.20 (dt, *J* = 7.6, 1.2 Hz, 1H), 7.15 (d, *J* = 7.4 Hz, 1H), 7.03 (dt, *J* = 7.5, 1.0 Hz, 1H), 6.90 (d, *J* = 7.8 Hz, 1H), 2.15 (sp, *J* = 6.8 Hz, 1H), 1.93 (q, *J* = 7.3 Hz, 2H), 1.01 (d, *J* = 7.0 Hz, 3H), 0.77 (d, *J* = 6.8 Hz, 3H), 0.60 (t, *J* = 7.4 Hz, 3H). ^13^C-NMR (150 MHz, CDCl_3_) δ 182.6, 141.6, 131.5, 127.5, 123.9, 122.0, 109.3, 57.6, 35.1, 28.2, 17.5, 17.2, 8.8. IR (KBr) ν 3164, 1717, 1668 cm^−1^. MS (ESI): 204.06 [M + H]^+^. EA Calcd. for C_13_H_17_NO (203.29): C, 76.81%; H, 8.43%; N, 6.89%. Found: C, 76.91%; H, 8.08%; N, 7.02%.

*3-Benzyl-3-isopropyl-1,3-dihydro-2H-indol-2-one* (**9k**). This compound was prepared according to the general procedure II using BuLi (7.85 mL, 12.55 mmol, 2.2 eq) in THF (8 mL), **8f** (1.00 g, 5.70 mmol) dissolved in THF (12 mL), and benzyl bromide (0.81 mL, 1.17 g, 6.84 mmol, 1.2 eq) in THF (2 mL). The product **9k** was obtained as colorless crystals (0.95 g, 63%), m.p. 133–134 °C (hexane–EtOAc). ^1^H-NMR (400 MHz, CDCl_3_) δ 7.38 (br s, 1H), 7.31 (d, *J* = 6.7 Hz, 1H), 7.12 (dt, *J* = 7.7, 1.2 Hz, 1H), 7.05–6.97 (m, 4H), 6.83 (dd, *J* = 7.4, 1.4 Hz, 2H), 6.61 (d, *J* = 7.7 Hz, 1H), 3.20 (d, *J* = 13.0 Hz, 1H), 3.17 (d, *J* = 13.0 Hz, 1H), 2.37 (sp, *J* = 6.8 Hz, 1H), 1.12 (d, *J* = 6.9 Hz, 3H), 0.83 (d, *J* = 6.8 Hz, 3H). ^13^C-NMR (150 MHz, CDCl_3_) δ 180.8, 141.1, 136.4, 130.7, 129.9, 127.6, 127.5, 126.2, 124.6, 121.8, 109.1, 58.6, 41.5, 35.5, 17.8, 17.4. IR (KBr) ν 3299, 1716, 1678 cm^−1^. MS (ESI): 266.16 [M + H]^+^. EA Calcd. for C_18_H_19_NO (235.36): C, 81.47%; H, 7.22%; N, 5.28%. Found: C, 81.26%; H, 7.16%; N, 5.25%.

### 3.4. General Procedure III for the Synthesis of Compounds ***21***

To a mixture of BuLi in hexane (4.45 mL, 7.13 mmol, 1.6 M) and THF (4.00 mL), the solution of the appropriate 3-alkyloxindole **8b** or **8f** (2.85 mmol) in THF (2 mL) was added dropwise at −78 °C, under argon atmosphere. The acetone-dry ice bath was removed, the reaction mixture was allowed to warm to room temperature and the apparatus was opened to the air. The stirring was continued for further 2 h, then the solvent was evaporated. The residue was taken up in water and extracted with EtOAc (3 × 15 mL). The organic layer was dried over MgSO_4_ and evaporated. The solid residue was triturated in DCM (2 mL). The white solid was filtered and dried. Analytical samples were obtained by recrystallization from the mixture of hexane–EtOAc.

*3-Hydroxy-3-isopropyl-1,3-dihydro-2H-indol-2-one* (**21a**) [56]. This compound was prepared according to the general procedure III. The product **21a** was obtained as colorless crystals (513 mg, 94%), m.p. 170–172 °C (hexane–EtOAc), lit. [56] m.p. 45–50 °C. ^1^H-NMR (400 MHz, DMSO-*d*_6_) δ 10.21 (br s, 1H), 7.23–7.18 (m, 2H), 6.95 (dt, *J* = 8.5, 1.0 Hz, 1H), 6.79 (d, *J* = 7.7 Hz, 1H), 5.77 (br s, 1H), 2.06 (sp, *J* = 6.9 Hz, 1H), 0.96 (d, *J* = 6.9 Hz, 3H), 0.63 (d, *J* = 6.8 Hz, 3H). ^13^C-NMR (100 MHz, DMSO-*d*_6_) δ 179.7, 142.4, 130.8, 128.9, 124.8, 121.5, 109.5, 78.5, 35.0, 16.4, 16.0. IR (KBr) ν 3350, 1702 cm^−1^. MS (ESI): 191.88 [M + H]^+^. Anal. Calcd. for C_11_H_13_NO_2_ (191.23): C, 69.09%; H, 6.85%; N, 7.32%. Found: C, 68.99%; H, 6.62%; N, 7.42%.

*3-Ethyl-3-hydroxy-1,3-dihydro-2H-indol-2-one* (**21b**) [57]. This compound was prepared according to the general procedure III. The product **21b** was obtained as colorless crystals (369 mg, 73%), m.p. 124–125 °C (hexane–EtOAc), lit. [48] m.p. 115–116 °C.

*5-Chloro-3,3-diethyl-1,3-dihydro-2H-indol-2-one* (**9l**). To a solution of **9d** (1.89 g, 10.00 mmol) in acetic acid (20 mL), sulfuryl chloride (1.62 mL, 2.69 g, 20.00 mmol) was added dropwise at 10–15 °C. The reaction mixture was stirred at 10 °C for 3 hours. The reaction mixture was poured onto ice-water (50 g) and stirred for one hour. The precipitate was filtered off, and washed with water until the pH was adjusted to 7. The crude product **9l** (2.09 g, 93%) was recrystallized from hexane–EtOAc to give **9l** (1.64 g, 73%) as colorless crystals, m.p. 166–167 °C (hexane–EtOAc). ^1^H-NMR (400 MHz, CDCl_3_) δ 9.33 (br s, 1H), 7.19 (dd, *J* = 8.2, 2.1 Hz, 1H), 7.10 (d, *J* = 2.0 Hz, 1H), 6.88 (d, *J* = 8.2 Hz, 1H), 1.97–1.89 (m, 2H), 1.84–1.76 (m, 2H), 0.65 (t, *J* = 7.4 Hz, 6H). ^13^C-NMR (100 MHz, CDCl_3_) δ 182.7, 140.2, 134.3, 127.8, 127.6, 123.4, 110.6, 55.4, 30.6, 8.6. IR (KBr) ν 3133, 1717 cm^−1^. MS (EI): 223 [M], 195, 194, 166, 159. EA Calcd. for C_12_H_14_ClNO (223.70): C, 64.43%; H, 6.31%; N, 7.32%; Cl, 15.85%. Found: C, 64.37%; H, 6.32%; N, 6.21%; Cl, 15.56%.

*5-Bromo-3,3-diethyl-1,3-dihydro-2H-indol-2-one* (**9m**) [32]. To a solution of **9d** (0.95 g, 5.00 mmol) in dioxane–water (10 mL–5 mL), a mixture of bromine (0.26 mL, 0.80 g, 5.00 mmol) and potassium bromide (1.19 g, 10.00 mmol) in water (10 mL) was added dropwise at 90 °C. After 10 min, water (5 mL) was added dropwise at 90 °C and crystals were precipitated. The reaction mixture was cooled with an ice bath, filtered off and washed with water and hexane. The product **9m** was obtained as colorless crystals (1.26 g, 94%), m.p. 165–166 °C (hexane–EtOAc), lit. [32] m.p. 164–165 °C. ^1^H-NMR (400 MHz, CDCl_3_) δ 8.76 (br s, 1H), 7.32 (dd, *J* = 8.2, 2.0 Hz, 1H), 7.21 (d, *J* = 2.0 Hz, 1H), 6.80 (d, *J* = 8.2 Hz, 1H), 1.96–1.86 (m, 2H), 1.81–1.71 (m, 2H), 0.63 (t, *J* = 7.4 Hz, 6H). ^13^C-NMR (100 MHz, CDCl_3_) δ 182.6, 140.7, 134.7, 130.5, 126.2, 115.1, 111.2, 55.4, 30.6, 8.6. IR (KBr) ν 3133, 1719, 1203, 812 cm^−1^. MS (EI): 267 [M], 239, 160, 159. EA Calcd. for C_12_H_14_BrNO (268.15): C, 53.75%; H, 5.26%; N, 5.22%; Br, 29.80%. Found: C, 54.00%; H, 5.40%; N, 5.11%; Cl, 29.37%.

*3,3-Diethyl-5-nitro-1,3-dihydro-2H-indol-2-one* (**9n**) [5]. To a solution of **9d** (11.06 g, 61.0 mmol) in cc. sulfuric acid (200 mL, 368 g, 3.75 mol), cc. nitric acid (2.6 mL, 3.93 g, 62.0 mmol) was added dropwise at 0 °C, the stirring was continued at room temperature for 2 hours. The precipitate was filtered off, washed with water until the pH was adjusted to 7 and dried. The product **9n** was obtained as pale brown crystals (12.40 g, 85%), m.p. 174–176 °C (EtOAc), lit. [5] m.p. 174–176 °C. ^1^H-NMR (400 MHz, DMSO-*d_6_*) δ 11.08 (br s, 1H), 8.20–8.14 (m, 2H), 7.05–7.03 (m, 1H), 1.94–1.89 (m, 2H), 1.79–1.73 (m, 2H), 0.51 (t, *J* = 7.4 Hz, 6H). ^13^C-NMR (100 MHz, DMSO-*d*_6_) δ 181.3, 149.4, 142.5, 133.3, 125.4, 119.1, 109.3, 54.4, 29.8, 8.6. IR (KBr) ν 1729, 1340 cm^−1^. MS (ESI): 233.15 (M − H)^−^. EA Calcd. for C_12_H_14_N_2_O_3_ (234.26): C, 61.53%; H, 6.02%; N, 11.96%. Found: C, 61.18%; H, 5.93%; N, 12.01%.

*3,3-Diethyl-2-oxo-2,3-dihydro-1H-indole-5-sulfonyl chloride* (**9o**). To sulfurochloridic acid (20 mL, 35.0 g, 300.0 mmol), **9d** (4.75 g, 25.0 mmol) was added at 15–20 °C, then the reaction mixture was heated at 60 °C for one hour. After cooling to room temperature the reaction mixture was added dropwise to ice. The precipitate was filtered off, washed with water until the pH was adjusted to 7, then it was washed with hexane and dried. The product **9o** was obtained as off-white crystals (6.62 g, 98%), m.p. 186–188 °C (EtOAc). ^1^H-NMR (600 MHz, CDCl_3_) δ 9.09 (br s, 1H), 7.98 (dd, *J* = 8.4, 2.0 Hz, 1H), 7.78 (d, *J* = 1.9 Hz, 1H), 7.13 (d, *J* = 8.3 Hz, 1H), 2.04–1.98 (m, 2H), 1.93–1.77 (m, 2H), 0.68 (t, *J* = 7.4 Hz, 6H). ^13^C-NMR (150 MHz, CDCl_3_) δ 182.3, 147.7, 138.3, 134.0, 128.7, 122.0, 109.8, 55.3, 30.5, 8.7. IR (KBr) ν 1729, 1366, 1175 cm^−1^. MS (EI): 287 [M], 259, 189, 161, 160, 159, 132, 130. EA Calcd. for C_12_H_14_ClNO_3_S (287.76): C, 50.09%; H, 4.90%; N, 4.87%; S, 11.14%; Cl, 12.32%. Found: C, 49.98%; H, 4.93%; N, 4.82%; S, 10.86%; Cl, 12.15%.

*5-Amino-3,3-diethyl-1,3-dihydro-2H-indol-2-one* (**9p**) [5]. To a solution of **9n** (800 mg, 3.42 mmol) in methanol (10 mL), activated palladium on charcoal (95 mg, 0.80 mmol, 10%) was added and the reaction mixture was placed into an autoclave (volume 70 mL). It was flushed with nitrogen, charged with hydrogen (20 bar) and heated to 70 °C while stirring. After 7 h the mixture was cooled to room temperature, filtered and the solvent was evaporated in vacuo. The residue was triturated in hexane, filtered off and dried. The product **9p** was obtained as off-white crystals (610 mg, 87%), m.p. 189–190 °C (EtOH), lit. [5] m.p. 188–190 °C. ^1^H-NMR (400 MHz, DMSO-*d*_6_) δ 9.89 (br s, 1H), 6.52 (d, *J* = 8.1 Hz, 1H), 6.44 (d, *J* = 2.2 Hz, 1H), 6.38 (dd, *J* = 8.1, 2.2 Hz, 1H), 4.66 (br s, 2H), 1.69–1.58 (m, 4H), 0.51 (t, *J* = 7.3 Hz, 6H). ^13^C-NMR (100 MHz, DMSO-*d*_6_) δ 180.3, 143.7, 132.9, 132.6, 112.7, 110.1, 109.4, 54.1, 30.2, 8.8. IR (KBr) ν 2963, 1684, 1496 cm^−1^. MS: 204 (M), 175, 157, 147, 132. EA Calcd. for C_12_H_16_N_2_O (204.27): C, 70.56%; H, 7.90%; N, 13.71%. Found: C, 70.05%; H, 7.68%; N, 13.65%.

*3,3-Diethyl-2-oxo-2,3-dihydro-1H-indole-5-sulfonamide* (**9q**). To a solution of **9o** (1.35 g, 5.00 mmol) in EtOH (50 mL), ammonium hydroxide solution (5 mL, 74.0 mmol, 25 *w*/*w*%) was added dropwise at 0 °C. The reaction mixture was stirred at room temperature for one hour. The volatile compounds were removed in vacuo and the crude product was recrystallized from acetic acid (25 mL) to give **9q** (0.80 g, 60%) as pale yellow crystals, m.p. 189–190 °C (EtOH). ^1^H-NMR (400 MHz, DMSO-*d*_6_) δ 10.76 (br s, 1H), 7.69 (dd, *J* = 8.1, 1.8 Hz, 1H), 7.64 (d, *J* = 1.8 Hz, 1H), 7.20 (s, 2H), 6.98 (d, *J* = 8.2 Hz, 1H), 1.83–1.71 (m, 4H), 0.53 (t, *J* = 7.3 Hz, 6H). ^13^C-NMR (100 MHz, DMSO-*d*_6_) δ 181.0, 145.9, 137.6, 132.5, 126.5, 120.8, 108.9, 54.1, 29.9, 8.7. IR (KBr) ν 3334, 1725, 1328, 1172 cm^−1^. MS (EI): 268 [M], 240, 159, 130. Anal. Calcd. for C_12_H_16_N_2_O_3_S (268.33): C, 53.71%; H, 6.01%; N, 10.44%; S, 11.95%. Found: C, 53.59%; H, 6.02%; N, 10.37%; S, 11.75%.

*N-tert-Butyl-3,3-diethyl-2-oxo-2,3-dihydro-1H-indole-5-sulfonamide* (**9r**). To a mixture of **9o** (2.90 g, 10.10 mmol) and sodium carbonate (1.20 g, 11.32 mmol) in THF (100 mL), *tert*-butylamine (2.4 mL, 1.68 g, 22.98 mmol) was added at room temperature. The reaction mixture was refluxed for 7 h, then the volatile compounds were removed in vacuo at 40 °C and hydrochloric acid (30 mL, 1.0 M) was added. The aqueous layer was extracted with EtOAc (3 × 15 mL), the combined organic layer was dried over MgSO_4_ and evaporated. The solid residue was triturated in DEE (5 mL), filtered off and dried. The product **9r** was obtained as colorless crystals (2.00 g, 61%), m.p. 202–203 °C (EtOH). ^1^H-NMR (400 MHz, CD_3_CN) δ 8.7 (br s, 1H), 7.72 (dd, *J* = 8.2, 2.0 Hz, 1H), 7.64 (dd, *J* = 1.8, 0.5 Hz, 1H), 7.01 (dd, *J* = 8.2, 0.3 Hz, 1H), 5.47 (s, 1H), 1.83 (q, *J* = 7.5 Hz, 4H), 1.13 (s, 9H), 0.56 (t, *J* = 7.5 Hz, 6H). ^13^C-NMR (100 MHz, CD_3_CN) δ 182.0, 146.9, 138.4, 134.1, 128.6, 123.0, 110.1, 55.6, 54.9, 31.3, 30.3, 9.00. IR (KBr) ν 3231, 1718, 1299, 1144 cm^−1^. MS (EI): 324 [M], 309, 252, 204, 188, 159. EA Calcd. for C_16_H_24_N_2_O_3_S (324.44): C, 59.23%; H, 7.46%; N, 8.63%; S, 9.88%. Found: C, 58.91%; H, 7.44%; N, 8.75%; S, 9.69%.

*3,3-Diethyl-5-(morpholin-4-ylsulfonyl)-1,3-dihydro-2H-indol-2-one* (**9s**). To a mixture of **9o** (2.88 g, 10.0 mmol) and sodium carbonate (1.59 g, 15.0 mmol) in THF (100 mL), morpholine (6.00 mL, 6.06 g, 69.6 mmol) was added at room temperature. The reaction mixture was refluxed for 5 h. The solvent was removed in vacuo at 40 °C and hydrochloric acid (30 mL, 1.0 M) was added. The aqueous layer was extracted with EtOAc (3 × 15 mL). The combined organic layer was dried over MgSO_4_ and evaporated. The solid residue was triturated in DEE (5 mL), filtered off and dried. The product **9s** was obtained as colorless crystals (2.58 g, 76%), m.p. 202–203 °C (EtOH). ^1^H-NMR (400 MHz, CDCl_3_) δ 8.78 (br s, 1H), 7.67 (dd, *J* = 8.2, 1.8 Hz, 1H), 7.51 (d, *J* = 1.7 Hz, 1H), 7.08 (d, *J* = 8.2 Hz, 1H), 3.78–3.75 (m, 4H), 3.00–2.97 (m, 4H), 2.02–1.96 (m, 2H), 1.88–1.83 (m, 2H), 0.65 (t, *J* = 7.4 Hz, 6H). ^13^C-NMR (100 MHz, CDCl_3_) δ 182.1, 145.8, 133.5, 128.7 (128.68, 128. 67), 122.8, 109.6, 66.1, 55.0, 46.0, 30.6, 8.7. IR (KBr) ν 1741, 1347, 1164 cm^−1^. MS (ESI): 339.14 [M + H]^+^. Anal. Calcd. for C_16_H_22_N_2_O_4_S (338.42): C, 56.79%; H, 6.55%; N, 8.28%; S, 9.47%. Found: C, 56.78%; H, 6.49%; N, 8.34%; S, 9.62%.

*5,7-Dichloro-3,3-diethyl-1,3-dihydro-2H-indol-2-one* (**9t**). To a solution of **9d** (3.78 g, 20.0 mmol) in acetic acid (40 mL), sulfuryl chloride (4.80 mL, 8.09 g, 60.0 mmol) was added dropwise at room temperature. The mixture was heated to 60 °C for 4 hours. It was cooled to room temperature, then the reaction mixture was poured onto ice–water (50 g). The precipitate thus obtained was filtered off, washed with water until the pH was adjusted to 7, then it was washed with hexane and dried to give **9t** (5.00 g, 97%). The crude product **9t** was recrystallized from water (80 mL) and EtOH (120 mL) to give **9t** (3.48 g, 13.48 mmol, 67%) as colorless crystals, m.p. 177–178 °C (water–EtOH). ^1^H-NMR (400 MHz, CDCl_3_) δ 8.08 (br s, 1H), 7.23 (d, *J* = 1.9 Hz, 1H), 7.01 (d, *J* = 1.8 Hz, 1H), 2.00–1.91 (m, 2H), 1.82–1.64 (m, 2H), 0.66 (t, *J* = 7.4 Hz, 6H). ^13^C-NMR (100 MHz, CDCl_3_) δ 180.4, 137.8, 135.2, 128.1, 127.4, 122.0, 115.1, 56.5, 30.7, 8.7. IR (KBr) ν 3183, 1725 cm^−1^. MS (EI): 259, 257 [M], 231, 230, 229, 228, 214, 200, 193, 165, 164. EA Calcd. for C_12_H_13_Cl_2_NO (258.14): C, 55.83%; H, 5.08%; N, 5.43%, Cl, 27.47. Found: C, 55.76%; H, 5.07%; N, 5.41%; Cl, 27.46%.

*5,7-Dichloro-3,3-diethyl-6-fluoro-1,3-dihydro-2H-indol-2-one* (**9u**). To a solution of **9g** (1.04 g, 5.0 mmol) in acetic acid (30 mL), sulfuryl chloride (2.00 mL, 3.34 g, 25.0 mmol) was added dropwise at room temperature. The mixture was heated to 70–80 °C for 1 h. It was cooled to room temperature, then the reaction mixture was poured onto ice–water (30 g). The precipitate thus obtained was filtered off, washed with water until the pH was adjusted to 7, then it was washed with hexane and dried. The product **9u** was obtained as colorless crystals (1.20 g, 87%), m.p. 192–193 °C (hexane–EtOAc). ^1^H-NMR (400 MHz, CDCl_3_) δ 9.05 (br s, 1H), 7.05 (d, *J* = 6.3 Hz, 1H), 2.00–1.94 (m, 2H), 1.81–1.74 (m, 2H), 0.67 (t, *J* = 7.4 Hz, 6H). ^13^C-NMR (100 MHz, CDCl_3_) δ 181.4, 153.5 (d, *J* = 248.3 Hz), 139.5 (d, *J* = 2.7 Hz), 128.6 (d, *J* = 4.2 Hz), 122.7, 115.0 (d, *J* = 18.7 Hz), 104.6 (d, *J* = 22.9 Hz), 56.2, 30.6, 8.6. IR (KBr) ν 3085, 1726, 1189, 770 cm^−1^. MS (EI): 277, 275 [M], 248, 246, 218, 211, 183. Anal. Calcd. for C_12_H_12_Cl_2_FNO (276.14): C, 52.20%; H, 4.38%; N, 5.07%; Cl, 25.68%. Found: C, 52.06%; H, 4.44%; N, 5.16%; Cl, 25.79%.

## 4. Conclusions

A systematic study of regioselective 3-alkylation reaction of N-unprotected-3-monosubstituted oxindoles was carried out after summarizing the literature of the numerous, albeit sporadic, precedents giving mostly unsatisfactory results. We have now elaborated an optimized method for the 3-alkylation of N-unsubstituted 3-alkyloxindoles by applying butyllithium as the base and alkyl bromides as the alkylating agents. The method has been extended to various alkyl groups in position 3 and various substituents on the aromatic ring. Introduction of new substituents into the aromatic ring of 3,3-diethyloxindole is disclosed. The formation of 3-hydroxylated side-products was investigated and the targeted synthesis of these compounds is also described. Owing to the presence of the unsubstituted nitrogen atom N1 in the title products (and the 3-hydroxy moiety in certain compounds), further functionalizations can also be carried out, thereby making these compounds valuable building blocks in synthetic organic or medicinal chemistry.

## Figures and Tables

**Table 1:** Alkylation reactions of 3-ethyloxindoles **8b**–**e** and 3-isopropyloxindole (**8f**) with various alkyl halides.

Entry	8	R^1^	R^3^	BuLi (eq)	R^2^X (eq)	9	9 Yield (%)	20 Yield (%)
1	**b**	Et	H	2.5	MeI (2.5)	**c**	28 ^a^	55 ^a^ (**20a**)
2	**b**	Et	H	2.2	MeI (1.2)	**c**	71	0
3	**b**	Et	H	2.5	EtI (2.5)	**d**	73	0
4	**b**	Et	H	2.2	EtBr (1.2)	**d**	90	0
5	**b**	Et	H	2.2	BnBr (1.2)	**e**	80	0
6	**c**	Et	5-Me	2.2	EtBr (1.2)	**f**	76	0
7	**d**	Et	6-F	2.2	EtBr (1.2)	**g**	77	0
8	**e**	Et	7-Me	2.2	EtBr (1.2)	**h**	66	0
9	**f**	*i*-Pr	H	2.5	MeI (2.5)	**i**	40 ^a^	35 ^a^ (**20b**)
10	**f**	*i*-Pr	H	2.5	MeI (1.2)	**i**	56	0
11	**f**	*i*-Pr	H	2.2	EtBr (1.2)	**j**	65	0
12	**f**	*i*-Pr	H	2.2	BnBr (1.2)	**k**	63	0

^a^ Isolated by flash chromatography.

**Table 2:** Various functionalizations of 3,3-diethyloxindole (**9d**) on the aromatic ring.

Entry	Reagents of Step 1 ^a^	Product of Step 1	R^4^	Yield of Step 1 (%)	Reagents of Step 2	Product of Step 2	R^5^	Yield of Step 2 (%)
1	SO_2_Cl_2_	**9l**	Cl	73	-	-	-	-
2	Br_2_, KBr	**9m**	Br	94	-	-	-	-
3	HNO_3_, H_2_SO_4_	**9n**	NO_2_	85	H_2_/Pd/C	**9p**	NH_2_	87
4	ClSO_3_H	**9o**	SO_2_Cl	98	NH_3_	**9q**	NH_2_SO_2_	60
5					*t*-BuNH_2_	**9r**	*t*-BuNHSO_2_	61
6					morpholine	**9s**	(morph)SO_2_	76

^a^ For Steps 1 and 2, and substituents R^4^, R^5^, see Scheme 11.
